# Brain responses during strategic online gaming of varying proficiencies: Implications for better gaming

**DOI:** 10.1002/brb3.1076

**Published:** 2018-07-18

**Authors:** Min Wang, Guangheng Dong, Lingxiao Wang, Hui Zheng, Marc N. Potenza

**Affiliations:** ^1^ Department of Psychology Zhejiang Normal University Jinhua China; ^2^ Institute of Psychological and Brain Sciences Zhejiang Normal University Jinhua China; ^3^ CAS Key Laboratory of Behavioral Science Institute of Psychology Beijing China; ^4^ Department of Psychiatry Department of Neurobiology Child Study Center, and National Center on Addiction and Substance Abuse Yale University School of Medicine New Haven Connecticut; ^5^ Connecticut Mental Health Center New Haven Connecticut

**Keywords:** automatic processing, brain responses, fMRI, functional connectivity, online gaming

## Abstract

**Background:**

Online gaming is a complex and competitive activity. However, little attention has been paid to brain activities relating to gaming proficiency.

**Methods:**

In the current study, fMRI data were obtained from 70 subjects while they were playing online games. Based on their playing, we selected 24 clips from each subject for three levels of gaming proficiency (good, poor, and average), with each clip lasting for 8 seconds.

**Results:**

When comparing the brain responses during the three conditions, good‐play trials, relative to poor‐ or average‐play trials, were associated with greater activation of the declive, postcentral gyrus, and striatum. In post‐hoc analyses taking the identified clusters as regions of interest to calculate their functional connectivity, activation of the declive during good‐play conditions was associated with that in the precentral gyrus and thalamus, and activation in the striatum was associated with that in the inferior frontal gyrus and middle frontal cortex.

**Conclusions:**

Taken together, findings suggest specific regional brain activations and functional connectivity patterns involving brain regions and circuits involved in sensory, motor, automatic and executive functioning and their coordination are associated with better gaming. Specifically, for basic functions, such as simple reaction, motor control, and motor coordination, people need to perform them automatically; for highly cognitive functions, such as plan and strategic playing, people need to engage more executive functions in finishing these works. The automatically processed basic functions spare cognitive resources for the highly cognitive functions, which facilitates their gaming behaviors.

## 1 INTRODUCTION

Online gaming has become an important recreational activity. It is estimated that teenagers spend an average of 31 hr per‐week online, mostly playing online games (McGonigal, 2011a,b). Teenagers will spend nearly ten thousand hours playing games by the time they are 21 years old, and collectively about 3 billion hours per week is spent on gaming around the world (McGonigal, 2011a,b). In one respect, online gaming may improve performance on visual, attentional, perceptual, and cognitive tasks (Anguera et al., 2013; Bejjanki et al., 2014; Li, Polat, Makous, & Bavelier, 2009) and has been associated with changes in neural functioning (West et al., 2015). On the other hand, excessive gaming has been linked to a variety of disorders, especially Internet gaming disorder, which may involve functional impairments and social, financial, and occupational difficulties (Dong, Wang, Du, & Potenza, 2017; Wang et al., 2016).

Online competitive gaming (e.g., eSports) has become an important social and economic phenomenon (Weiss & Schiele, 2013). Like traditional sporting events, worldwide online gaming competitions (e.g., world cyber arenas for Worlds of WarCraft, Defense of the Ancients, League of Legends) are popular, with some competitions offering winners several million dollars (http://www.esportsearnings.com). Specific types of gaming, especially strategic forms, often involve complex, cooperative, and competitive behaviors. Competitive gaming often requires that players monitor fast‐paced and concurrent visual and auditory stimuli, using proper strategies to react quickly to competitors and their behaviors, and in doing so, individuals playing competitive games often need to react rapidly and switch flexibly between tasks while holding information in working memory (Bejjanki et al., 2014; Richlan, Schubert, Mayer, Hutzler, & Kronbichler, 2018; Wang, Zhu, Qi, Huang, & Li, 2017; West et al., 2015). Thus, this process requires the involvement of brain regions involved in multiple processing domains (e.g., visual, attention, motor control, working memory, strategic planning) (Katsyri, Hari, Ravaja, & Nummenmaa, 2013; Stockdale, Morrison, Palumbo, Garbarino, & Silton, 2017). Coordination of brain processes in these domains that span basic and higher level executive functioning may be necessary for optimizing gaming.

Although several studies have investigated brain responses to gaming and their relationships to cognitive functioning (Bejjanki et al., 2014; West et al., 2015), however, few have investigated brain activities relating to gaming proficiency (that is, good vs. average or poor gaming). Focusing on this issue could provide an improved understanding of brain responses involved in gaming performance, and allow for exploration of brain features that are responsible for good/bad/average gaming. This may also help with understanding what makes players perform well or poorly. For example: Are there any key regions that are responsible for good gaming, or what happened when they were playing poorly? In addition, this could potentially help with improving gaming abilities. To examine this issue, we collected gaming data from when individuals were gaming. Thereafter, we selected good, poor, or average play clips based on gaming performance and investigated specific regional brain activations and patterns of functional connectivity associated with better gaming.

Brain regions previously implicated in gaming include those within sensory and motor‐control‐related brain systems (Bejjanki et al., 2014; Kuhn & Gallinat, 2014). Consistently, competitive online gaming often requires players to coordinate sensory and motor control to accomplish their tasks and win against competitors (Anderson, Bothell, Fincham, & Moon, 2016; Sohn, Lee, Kwak, Yoon, & Kwon, 2017). Brain regions, such as the cerebellum (involved in processing automatic or highly learned motoric behaviors and coordinating control), the thalamus (a neurocircuitry hub coordinating auditory, visual, and somatosensory functions), and the brain stem (with ascending and descending pathways involved in sensory and motoric processes), have been implicated in sensory and motor processes and their coordination (Katsyri et al., 2013; Koepp et al., 1998). In the current study, we hypothesized that good gaming would engage sensory‐ and motor‐control‐related brain regions (e.g., occipital cortex and pre and postcentral gyral regions, respectively) and those involved in coordinating their functions (e.g., the thalamus).

People, particularly males, often enjoy gaming, finding it interesting and motivating, and these features may be related to striatal dopamine release (Katsyri et al., 2013; Koepp et al., 1998). The successful achievement of specific gaming goals (e.g., eliminating one's opponents or avoiding getting eliminated oneself) may trigger positive emotions (Nummenmaa & Niemi, 2004), which may be related to dopamine release (Koepp et al., 1998) and hemodynamic activations in the striatum (Hoeft, Watson, Kesler, Bettinger, & Reiss, 2008; Katsyri et al., 2013). Neuroimaging studies suggest that gaming engages key motivational systems of the brain (Katsyri et al., 2013).

Besides potential relationships to motivation and reward, response selection and sensorimotor coordination may also be related to striatal function during gaming (Koepp et al., 1998). In a recent imaging study involving subjects scanned before and after substantial gaming practice, better players showed greater activation in the right dorsal striatum (Anderson et al., 2016). Another study demonstrated selective striatal sensitivity to self‐acquired rewards, with striatal responses to repeated acquisition of rewards contingent on game‐related successes contributing to the motivational aspects of gaming (Katsyri et al., 2013). Good players should have strong motivations to win, and at the same time, they should have well coordinated motor and sensory systems. Thus, we hypothesized that good gaming (as compared to poor gaming) would involve greater striatal activation.

## 2 METHODS

### 2.1 Ethics

The experiment conforms to The Code of Ethics of the World Medical Association (Declaration of Helsinki). The Human Investigations Committee of Zhejiang Normal University approved the research. All participants provided written informed consent before scanning.

### 2.2 Participants

Valid data were collected from 70 university students (male, 45; female, 25; age (mean ± *SD*): 22.3 ± 2.1 years) who were recruited through advertisements. All subjects were free of psychiatric disorders (including major depression, anxiety disorders, schizophrenia, and substance‐dependence disorders) as assessed by the MINI International Neuropsychiatric Interview (Lecrubier et al., 1997). All subjects were familiar with the game *League of Legends* (LOL, Riots Games). Subjects needed to have played this game for more than 2 years and still be playing it for more than 5 times per week at the time of study participation.

### 2.3 Imaging data collection

MRI data were acquired using a Siemens Trio 3T scanner (Siemens, Erlangen, Germany) in the MRI center of the East‐China Normal University. Structural images were collected using a T1‐weighted three‐dimensional spoiled gradient‐recalled sequence covering the whole brain (176 slices, repetition time = 1,700 ms, echo time TE = 3.93 ms, slice thickness = 1.0 mm, skip = 0 mm, flip angle = 15, inversion time = 1,100 ms, field of view = 240×240 mm, in‐plane resolution = 256×256). Functional MRI was performed on a 3T scanner (Siemens Trio) with a gradient‐echo EPI T2* weighted sensitive pulse sequence in 33 slices (interleaved sequence, 3 mm thickness, TR = 2,000 ms, TE = 30 ms, flip angle 90°, field of view 220×220 mm^2^, matrix 64×64). The scan lasted for about 40 min (7 min for structural images, 30 min for gaming, and 3 min for preparation steps).

### 2.4 Task and conditions

Subjects were instructed to play one round of the “League of Legends” (a popular strategic online game) in the scanner. Their gaming behaviors were captured via screen recording. Afterwards, a researcher familiar with the game analyzed the gaming videos and selected 45 clips (15 8‐s periods each of good, poor or average play) from the screen recordings. The criteria used in selecting these clips included for good/poor play, respectively: (a) the use of proper/improper strategies; and, (b) correct/incorrect reactions to opponents’ behaviors. Trials that were difficult to assign to good or poor were considered average.

After the first round of selection, six players familiar with the game were asked to rate these clips according to their judgment. The rating used a 9‐point Likert questionnaire, from 1 (really poor) to 9 (really good). We selected the highest/lowest scored 8 clips for good/poor gaming, and the 8 clips that scored around 5 were considered as average (Cronbach's *α* = 0.705). The average clips were used as a baseline in the current study.

To minimize potential influences of strong emotions that may influence results, we strove to select clips that did not include situations that seemed likely to evoke strong emotions, such as killing, winning, or losing phenomena. We focused on clips that included arguably more neutral processes of gaming, such as organizing, preparing and using strategies.

### 2.5 Image preprocessing and analysis in task‐state data

The task‐based fMRI data were analyzed using SPM12 (Statistical Parametric Mapping) (http://www.fil.ion.ucl.ac.uk/spm). Images were preprocessed using standard steps, as follows. Images were sliced‐timed, reoriented, and realigned to the first volume. Then, T1‐co‐registered volumes were normalized to an SPM T1 template and smoothed using a 6‐mm FWHM Gaussian kernel spatially.

A general‐linear‐model (GLM) approach was used to examine blood oxygen level dependence (BOLD) signals related to the three event types (good, poor, average). Each condition consisted of 5 blocks, and each block lasted for 8 seconds, as described in the “task and condition” section. Six head motion parameters were included in the GLM. To improve the signal‐to‐noise ratio, a high‐pass filter (cut‐off period of 128 s) was used to remove low frequency noise. We took the average condition as the baseline and investigated the specific features associated with better gaming proficiency in good‐poor, good‐average and average‐poor contrasts. AlphaSim correction (an approach to correct for multiple comparisons using Monte Carlo simulations) (updated version; *p *<* *0.01, clusters >120 voxels) was used in the current study (https://afni.nimh.nih.gov/pub/dist/doc/program_help/3dClustSim.html).

### 2.6 Functional connectivity analysis

The functional data were modeled according to the onset time of clips. Eight clips in the three different conditions were taken as eight runs of scan. Then, the data were preprocessed using DPARSF4.0 (http://rfmri.org/DPARSF) and REST (http://www.rest.restfmri.net). First, all data were slice‐timed, reoriented, and realigned to the first volume (preprocessing). No subjects were excluded based on the criteria of translational movement <2.0 mm and <2.0° rotation. Next, images were normalized using EPI templates and then smoothed. Finally, the data were filtered with a temporal band pass between 0.01 and 0.08 Hz.

Based on the contrast of good versus poor conditions, we selected three significant clusters as the regions of interest (ROI) for further connectivity analyses: the declive of the cerebellum, the striatum, and the precentral gyrus (this ROI was selected from the good‐average comparison). For each ROI, a seed reference time course was obtained by averaging the time series of all voxels in the ROI. Then, Pearson's correlation analysis was performed between the seed reference time course and the time series from the whole brain in a voxel‐wise way, with the global signal, white‐matter signal, cerebrospinal‐fluid signal, keyboard pressing times during these period and the six head‐motion parameters included as nuisance covariates. Finally, the resultant correlation coefficients were transformed into *z*‐scores using Fisher's transformation for better satisfaction of normality. Finally, we used the two‐sample *T*‐test to compare the difference in the functional connectivity in different comparisons (good‐poor, good‐average, and average‐poor).

## 3 RESULTS

### 3.1 GLM results

**Figure 1:**
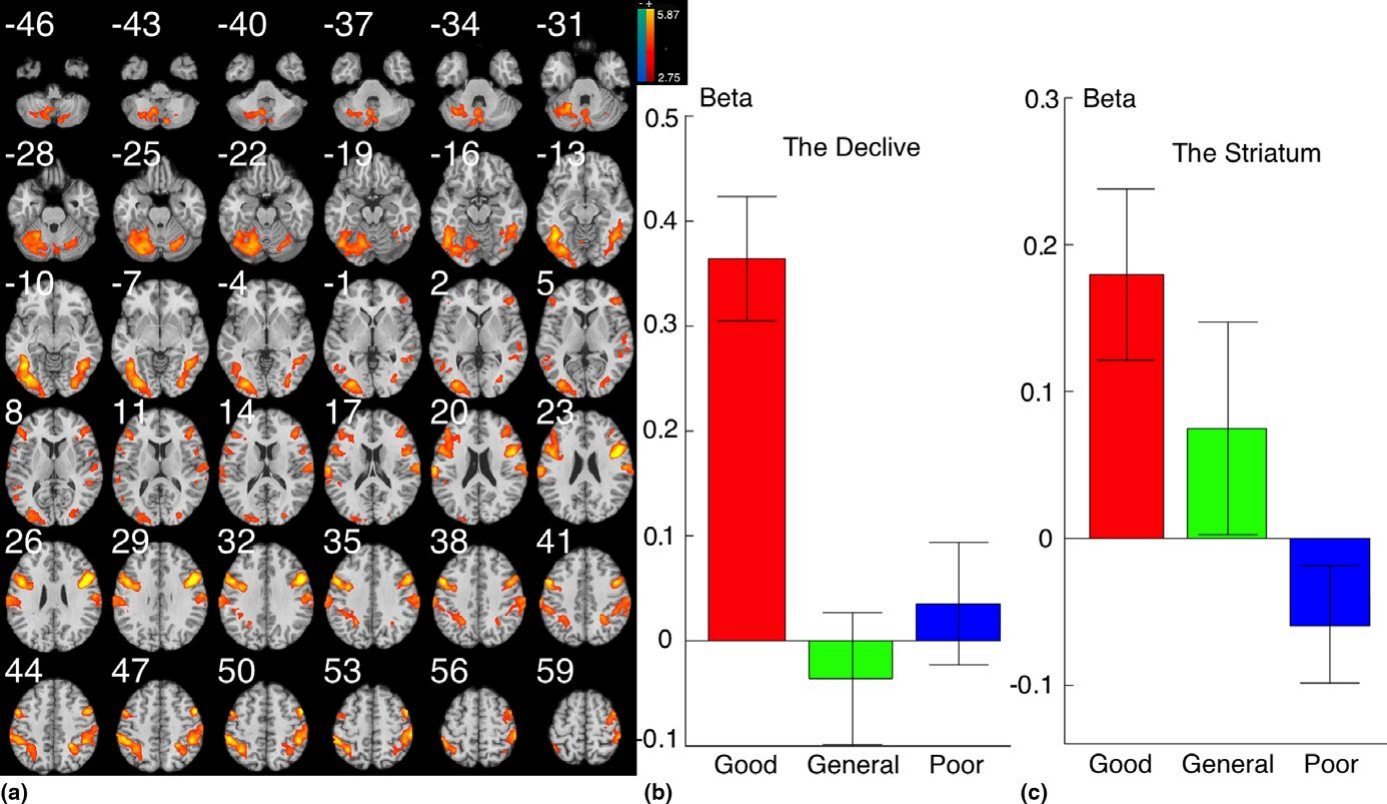
Brain regions surviving the good‐poor comparison. (a) Good gaming is associated with higher brain activations in the occipital gyrus, the declive, the pre and postcentral gyrus and the striatum when compared to poor gaming (*p *<* *0.01, cluster size >120). (b) Beta weights indicating activation of the declive in the different conditions. Declive activation was greatest in the good condition. (c) Beta weights indicating activation of the striatum in the different conditions. Striatal activation was greatest in the good condition

As compared with poor‐play trials, good‐play trials were associated with greater brain activations in the declive, bilateral striatum, bilateral postcentral gyrus, and the occipital gyrus (Figure 1a; Table 1). The good‐average comparison (Supporting Information Figure S1; Table 1) and the average‐poor comparison (Supporting Information Figure S2; Table 1) are displayed in the supplementary materials. The extraction of beta‐weights from the declive (Figure 1b) and the striatum (Figure 1c) indicates that the difference between the good and poor conditions was related to increased brain activation in good‐play trials.

**Table 1:** Regional brain responses in different comparisons among good, poor, and average conditions

Cluster number	*x, y, z* a	Peak intensity	Cluster size^b^	Region^c^	Brodmann's area
Good‐Poor					
1	24, −21, 27	5.143	786	R Striatum	
2	−21, −15, 15	4.254	352	L Striatum	
3	39, −48, −3	5.790	1451	L R Occipital gyrus	19, 36
4			847	L R Declive	
5	27, −54, 30	4.176	194	R Postcentral Gyrus	5
6	−18, −57, 39	4.281	288	L Postcentral Gyrus	5, 7
Good‐Average					
1	48, 9, 30	4.852	519	R Inferior Frontal Gyrus	44, 45, 47
2	42, 33, 12	4.126	143	R Medial Frontal Gyrus	44, 45, 47
3	−51, 6, 39	4.672	597	L Precentral Gyrus	4
4	48, −39, 48	5.033	1209	R Inferior Parietal Lobule	39
5	−51, −36, 51	4.725	634	L Inferior Parietal Lobule	39
6	−42, −57, −12	5.627	1636	L Middle Occipital Gyrus	18
Average‐Poor					
1	63, −24, 33	4.326	374	R Inferior Parietal Lobule	39
2	−54, 9, 30	3.983	152	L Precentral Gyrus	4
3	54, 6, 48	4.142	165	R Precentral Gyrus	4

^a^Peak MNI Coordinates; ^b^Number of voxels. *p *<* *0.01, cluster size>120 contiguous voxels. Voxel size = 3×3×3; ^c^The brain regions were referenced to the software Xjview (http://www.alivelearn.net/xjview8) and verified through comparisons with a brain atlas.

### 3.2 Functional connectivity between different brain regions

**Figure 2:**
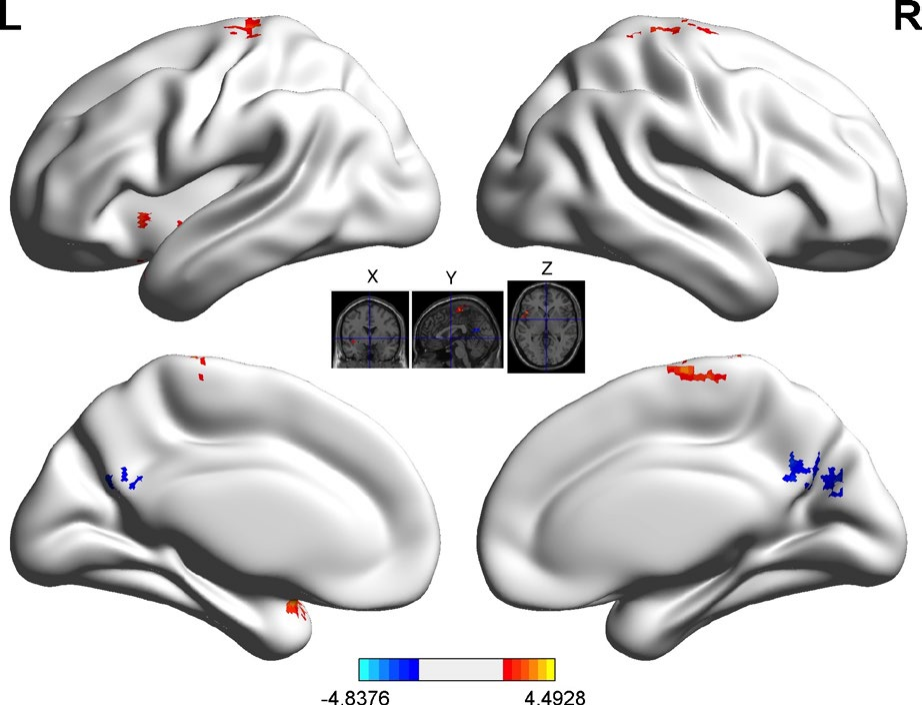
Functional connectivity between the declive and other brain regions in the good‐poor comparison (*p *<* *0.01, cluster size >120)

We selected three ROIs according to our a priori hypotheses and the results from the good‐poor and good‐average comparisons: the declive (in the cerebellum), the striatum, and the precentral gyrus. When selecting the declive as an ROI, higher functional connectivity in good‐play trials was found between the declive and bilateral inferior parietal lobe, and lower functional connectivity in poor‐play trials was observed with the middle frontal gyrus (Figure 2; Supporting Information Figures S3 and S4; Table 2). Other comparisons between good‐average and poor‐average trials are presented in the Supporting Information Figures S5 and S6.

**Table 2:** Functional connectivity differences between good and poor gaming

Cluster number	*x, y, z* a	Peak intensity	Cluster size^b^	Region^c^	Brodmann's area
Taking the Declive as ROI					
1	−30, 3, −18	3.649	142	L Superior Temporal Gyrus/Inferior Parietal Lobe	41, 42, 44, 45
2	12, −72, 27	−3.462	125	R Medial Frontal Gyrus	7
3	−21, −27, 60	3.764	189	L Precentral Gyrus	4, 6
Taking the Precentral Gyrus as ROI					
1	0, 27, −21	−5.278	136	Orbital Frontal Gyrus	8, 9
2	3, 27, 39	4.077	497	Superior Frontal Gyrus/Medial Frontal Gyrus	4, 6, 8, 9
Taking the Striatum as ROI					
1	9, 0, 54	3.632	151	R Anterior Cingulate Cortex	6, 23, 24
2	39, −24, 48	3.884	185	R Middle Frontal Gyrus	4
				R Inferior Frontal Gyrus	
3	−24, 6, 51	3.749	197	L Postcentral Gyrus	1, 2, 3

^a^Peak MNI Coordinates; ^b^Number of voxels. AlphaSim FWE correction *p* < 0.01with 120 contiguous voxels. Voxel size = 3×3×3; ^c^The brain regions were referenced to the software Xjview (http://www.alivelearn.net/xjview8) and verified through comparisons with a brain atlas.

**Figure 3:**
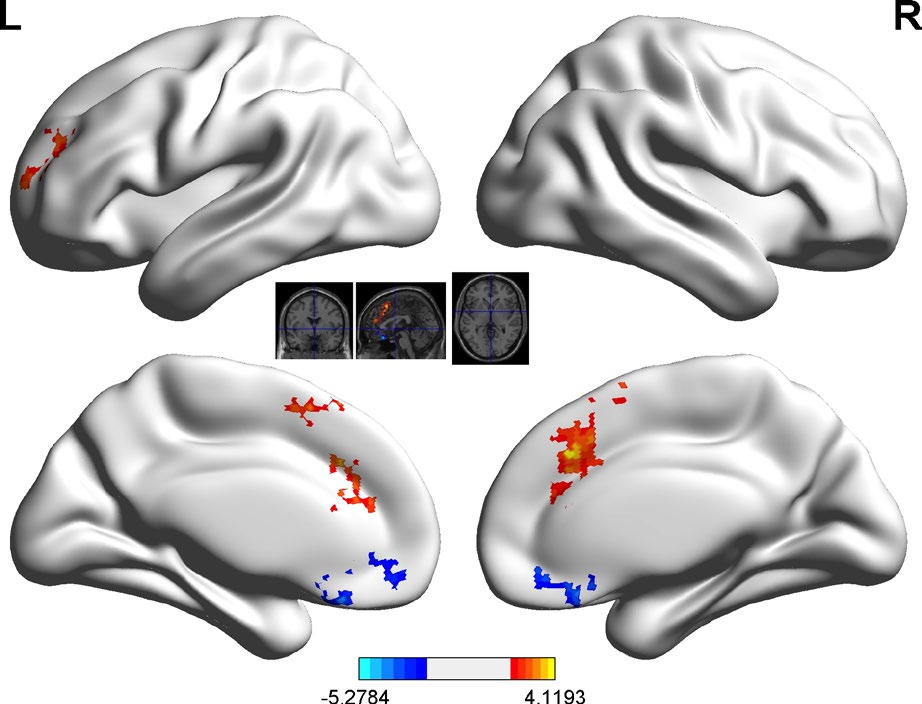
Functional connectivity between the prefrontal gyrus and other brain regions in the good‐poor comparison (*p *<* *0.01, cluster size >120)

Using the precentral gyrus as a seed, activity in the precentral gyrus correlated positively with activity in the middle prefrontal gyrus and inversely with activity in the orbital frontal cortex during good‐play trials (Figure 3; Table 2). Functional connectivity with the precentral gyrus in the good‐average and average‐poor trials is shown in the Supporting Information Figures S7 and S8.

**Figure 4:**
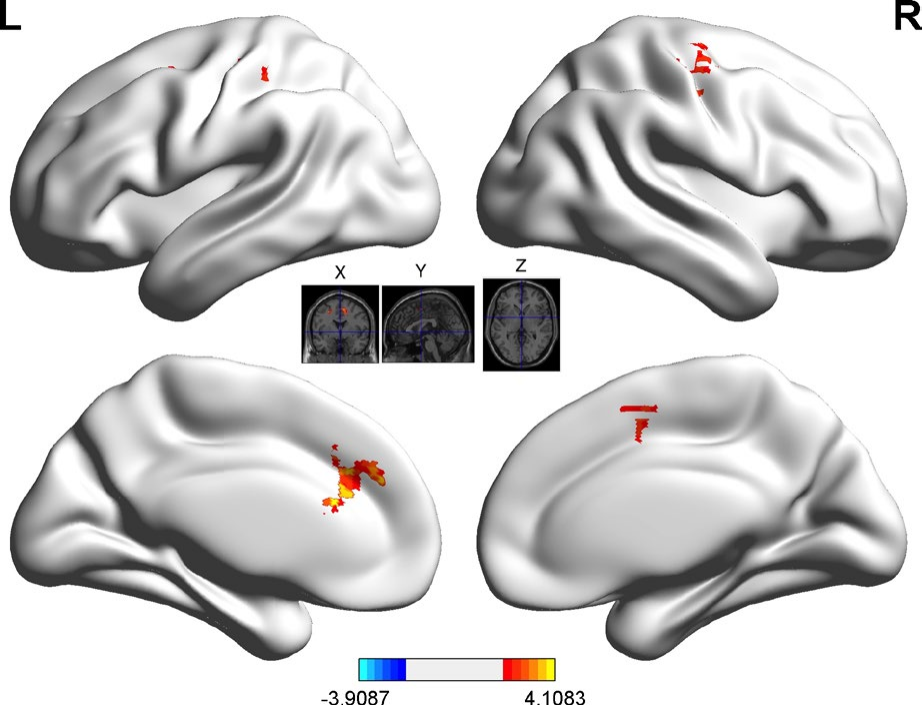
Functional connectivity between the striatum and other brain regions in the good‐poor comparison (*p *<* *0.01, cluster size >120)

Using the striatum as a seed, activity in the striatum correlated positively with that in the middle frontal gyrus and bilateral anterior cingulate cortex (Figure 4; Table 2). Functional connectivity with the striatum in the good‐general and general‐poor comparisons is shown in the Supporting Information Figures S9 and S10.

## 4 DISCUSSION

### 4.1 Good gaming may involve learned “automatic” or habitual processing

In the current study, we examined brain activity underlying different proficiencies of gaming. Good‐play trials were associated with greater brain activations in the declive, with findings driven by increased activation in good‐play trials. When taking the declive as a ROI, activity in the declive was associated with that in the precentral gyrus and the middle prefrontal gyrus, in good as compared with poor or average gaming trials. The declive is part of the cerebellum and has been implicated in automatic motor control, motor adaptation and the acquisition of new motor skills (Ramnani, 2014; Takakusaki, 2017). It may influence motoric control by integrating sensory and cortical signals and projecting signals to the motor areas of the cerebral cortex and brainstem (Molinari & Leggio, 2013). In the current study, good gaming engaged greater involvement of the declive, and its activity was correlated with that in the precentral gyrus, a motor control area (Georgopoulos & Carpenter, 2015). These findings suggest that good gaming may involve activations of regions and circuits involved in the coordination of motor processes. One speculative possibility is that gaming may involve aspects of automatic processing. Such an interpretation would be consistent with findings from sports studies in which automatic processing may be accompanied by improved performance, with extra attention potentially disrupting optimal performance (Wu et al., 2015; Wulf & Prinz, 2001). Studies have also suggested that during a competitive task, more attention to performance, leading to less automaticity, may disrupt a well‐practiced skill (Wulf & Prinz, 2001). Other findings suggest that when a movement becomes automatic, neural efficiency is increased by the strengthening of connections within specific neural networks (Balsters & Ramnani, 2011; Wu et al., 2015). Long‐term practice leading to good gaming may in part involve overlearned motor sequences. In other words, gaming behaviors have become more automatic following repetitive playing. In addition, competitive gaming may require rapid and correct responses. Such responses may engage brain regions and systems involved in more automatic responses. If this interpretation is correct, it would have implications for the neural underpinnings of practice leading to improved gaming proficiency. While currently speculative, additional research should examine the degree to which brain regions and networks involved in automatic or habitual responding may contribute to gaming proficiency.

### 4.2 Good gaming may involve coordination between sensory and motor‐control systems

As hypothesized, good‐play trials were associated with greater activation of the precentral gyrus, postcentral gyrus, thalamus, and occipital gyrus. During good‐play as compared to poor‐play trials, functional connectivity was relatively increased between the precentral gyrus and the occipital gyrus and between the precentral gyrus and thalamus. The precentral gyrus controls motoric behaviors (Georgopoulos & Carpenter, 2015), whereas the occipital gyrus processes visual information (Janssen, Verhoef, & Premereur, 2018). The thalamus represents a connectivity hub for information processing, including the relaying of sensory and motor signals to the cerebral cortex (Cassel et al., 2013). The greater regional activations in the visual system, motor system, and the thalamus, and the enhanced functional connectivity among these brain regions, in the good‐playing trials suggest better coordination of visual, sensory and motor control during good as compared to poor gaming. These findings seem consistent with the notion that playing online games well involves relatively greater activation of sensory and motor systems and their coordination (Bejjanki et al., 2014; West et al., 2015).

### 4.3 Good gaming may involve the coordination of executive cognitive functions

As hypothesized, good as compared with poor play was associated with greater activation in the striatum, which has been suggested to be important in motivation, response selection and sensorimotor coordination (Dong, Wang, & Potenza, 2016; Koepp et al., 1998). In addition, stronger functional activity was found between the striatum and the middle frontal gyrus, inferior frontal gyrus and precentral gyrus. While the striatum has been implicated in reward, motivation and learning, it has also been linked to the use of cognitive strategies (Iaria, Petrides, Dagher, Pike, & Bohbot, 2003; West et al., 2015) and stimulus‐response learning, which involves making a particular action when facing a specific environmental stimulus (Lerch et al., 2011). Koepp et al. hypothesized that the ventral striatum related to affective aspects of gaming, and dorsal striatal function was linked to response selection and sensorimotor coordination (Koepp et al., 1998). Greater activation in the striatum has also been correlated with better gaming skills (Anderson et al., 2016). Thus, from this point of view, the higher striatal activation in good versus poor gaming might suggest a role for this brain region in better response selection and sensory coordination, although additional research is needed to investigate this possibility. During good versus poor gaming, the striatum was found to be more strongly functionally connected with brain regions previously implicated in executive control (middle frontal gyrus), motor control (precentral gyrus), and decision‐making (inferior frontal gyrus) (Brown, 2011; Crone & Steinbeis, 2017; Gourley & Taylor, 2016; Posner & Rothbart, 1998). Based on the current literature on the role of the striatum in gaming, multiple explanations are possible, including both proposed by Koepp et al. (1998), with the latter (relating to response selection and sensorimotor coordination) perhaps fitting better with the current findings. Future studies should design specific tasks to examine the precise roles for the striatum and related circuits during gaming and as linked to gaming proficiency.

Another interesting result involves the implication of the middle frontal gyrus and inferior frontal gyrus. First, the good‐play as compared to the average‐play trials was associated with greater brain activations in the middle frontal gyrus and inferior frontal gyrus. In addition, during good‐play trials, the prefrontal gyrus was functionally connected with the middle frontal gyrus, inferior frontal gyrus, and anterior cingulate cortex, suggesting important roles for these regions during good gaming. The middle frontal gyrus and anterior cingulate cortex have been implicated in executive control; for example, individuals with frontal lesions exhibit problems with goal‐directed behaviors, especially in novel tasks involving control processing (Brown, 2011; Crone & Steinbeis, 2017; Gourley & Taylor, 2016; Posner & Rothbart, 1998). The inferior frontal gyrus has been implicated in decision‐making (Jimura, Chushak, Westbrook, & Braver, 2017; Wang et al., 2016) and inhibitory control (Bari & Robbins, 2013; Shao, Zhang, & Lee, 2015). The inferior frontal gyrus is also a critical area for action implementation based on value information, through connections with limbic areas via the insula and with motor cortices (Bari & Robbins, 2013; Shao et al., 2015). Together, the findings that activations in and functional connectivity between regions previously implicated in executive control are consistent with good gaming being linked to better planning and strategic decision‐making, although further testing of this possible interpretation is warranted.

### 4.4 Limitations

Several limitations should be noted. First, there are no standard criteria in defining good/poor/average game‐playing. Thus, we used a ranking method in separating different conditions based on two stages of clip review. Second, there are eight clips and each clip lasts for 8 seconds in one condition, the data from specific conditions are limited. Thus, we studied a larger sample subjects (*n* = 70) to help mitigate this limitation. Third, to keep the game‐playing process natural, no behavioral indexes (response time, accuracy rates) were recorded during scanning, which limits the conclusions that may be drawn, particularly between brain and behavior responses.

## 5 CONCLUSIONS

The current study suggests that better gaming proficiency may be associated with activation in brain regions and circuitry involved in automatic or highly learned motoric processing and coordination of sensory, motor and executive functioning. These aspects may at first seem contradictory but suggest that proficient gaming is a complex process involving multiple brain regions and circuits, including those involved in rapid, automatic responding as well as those involved in decision‐making and planning. Specifically, for basic functions, such as simple reaction, motor control, and motor coordination, people need to perform them automatically; for highly cognitive functions, such as plan and strategic playing, people need to engage more executive functions in finishing these works. We speculate that the automatically processed basic functions spare cognitive resources for the highly cognitive functions, which facilitates their gaming behaviors.

The current study provide evidence on the brain features of varying proficiencies, which deepened our understanding about the brain features on gaming, especially brain regions and features that responsible for good/bad/average gaming. Additional research is needed to confirm and extend these results, and examine the extent to which they generalize to groups of different ages, cultural backgrounds, and types of gaming.

## AUTHOR CONTRIBUTION

Guangheng Dong designed the task and wrote the first draft of the manuscript, Min Wang, Lingxiao Wang, Hui Zheng collected and analyzed the data, prepared the tables and figures. Marc Potenza contributed in editing, interpretation and revision processes. All authors contributed to and have approved the final manuscript.

## FINANCIAL DISCLOSURES

Guangheng Dong, Min Wang, Lingxiao Wang, and Marc Potenza report no conflicts of interest. Dr. Potenza has consulted for Shire, INSYS, Rivermend Health, Opiant/Lakelight Therapeutics, and Jazz Pharmaceuticals; has received research support from the Pfizer, Mohegan Sun Casino and the National Center for Responsible Gaming; has participated in surveys, mailings or telephone consultations related to drug addiction, impulse‐control disorders, or other health topics; has consulted for gambling and legal entities on issues related to impulse‐control/addictive disorders; provides clinical care in a problem gambling services program; has performed grant reviews for the National Institutes of Health and other agencies; has edited journals and journal sections; has given academic lectures in grand rounds, CME events and other clinical or scientific venues; and has generated books or book chapters for publishers of mental health texts.
