# Supplementary material for: Brain responses during strategic online gaming of varying proficiencies: Implications for better gaming
Source: Brain Behav. 2018 Jul 18;8(8):e01076. doi: 10.1002/brb3.1076 (PMC6085917; doi:10.1002/brb3.1076)
Supplement: Supplementary file 1 [file BRB3-8-e01076-s001.docx]

**Supplementary files to:**

**Brain responses during strategic online gaming of varying proficiencies: Implications for better gaming**

**This file includes:**

10 Figures

6 Tables


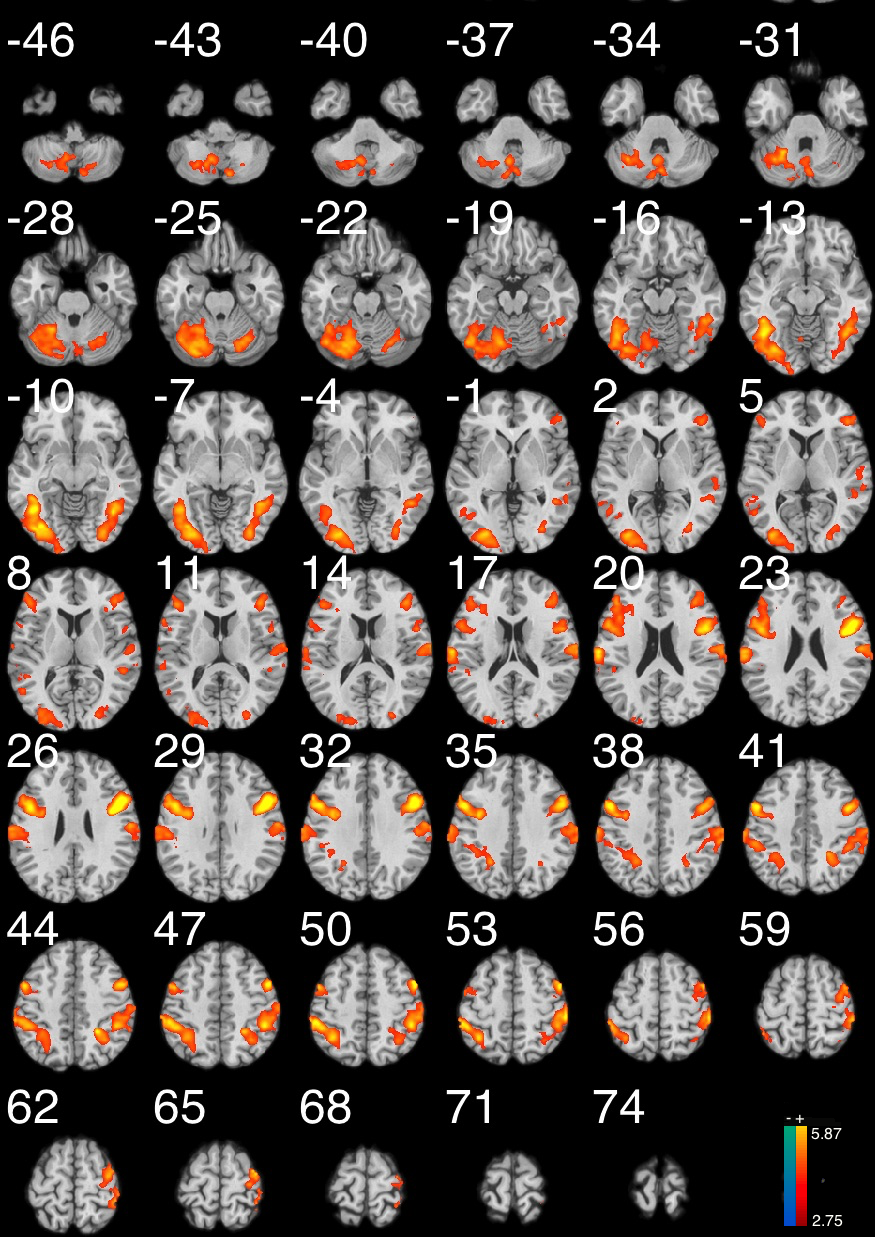


**Supplementary Figure 1: Brains regions surviving the good-average comparison.** The good - average comparison showed that the bilateral middle frontal gyrus, bilateral inferior frontal gyrus, the parietal lobe and bilateral occipital gyrus were activated to a greater extent during good-play trials (*p*<0.01, cluster size>120 contiguous voxels).

.


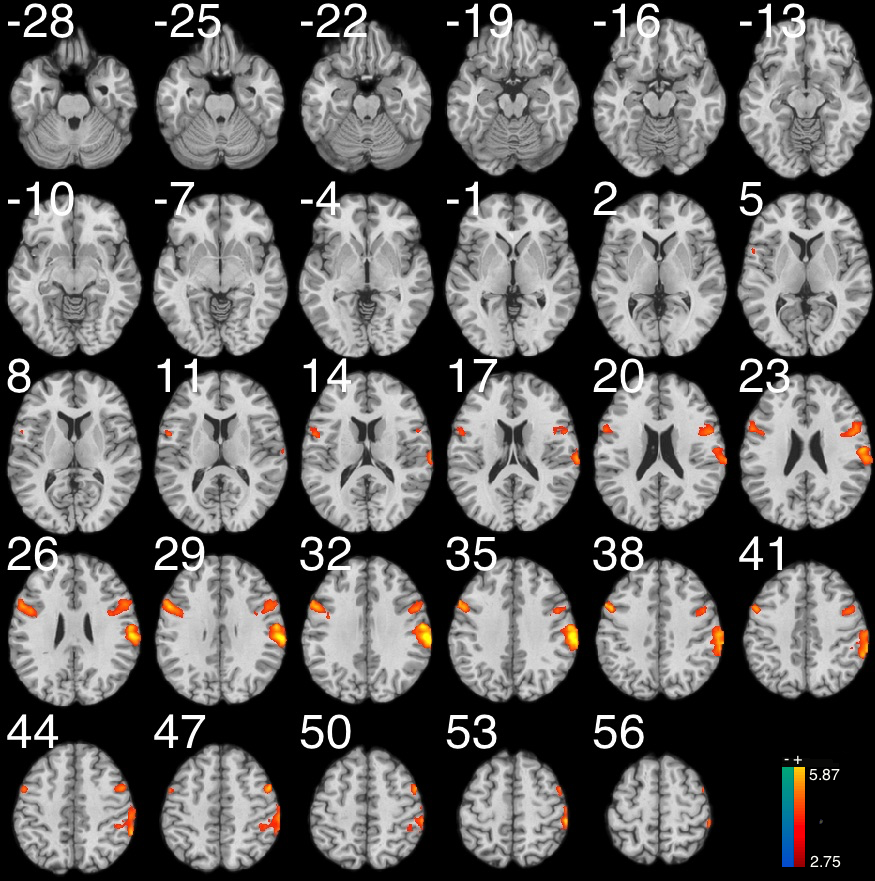


**Supplementary Figure 2: Brains regions surviving the poor-average comparison.**

The average - poor comparison showed the right parietal lobe and the bilateral precentral gyrus were activated to a greater degree in the average trials (*p*<0.01, cluster size>120 contiguous voxels).


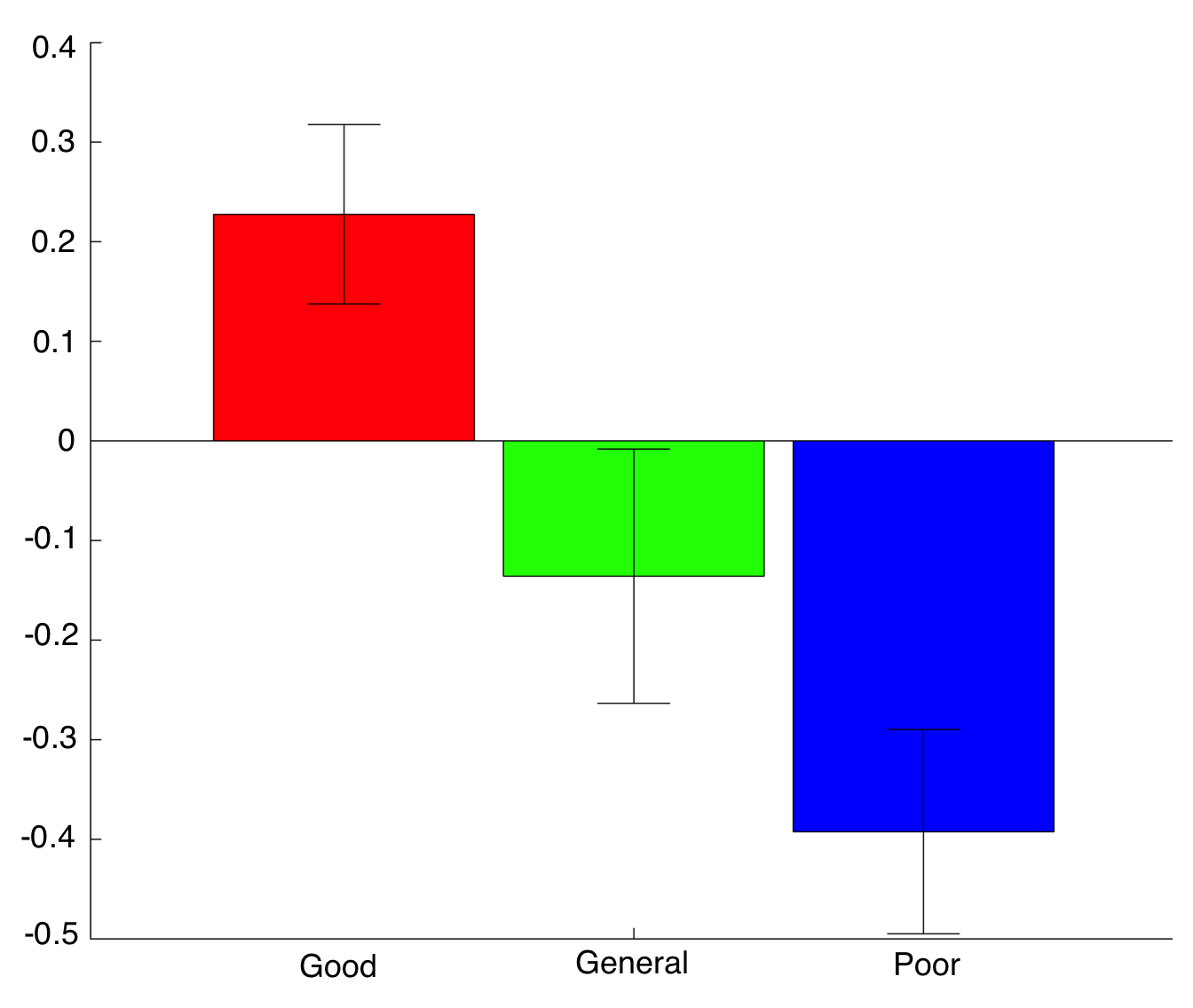


**Supplementary Figure 3:** Beta weights representing activation of the middle frontal cortex in the different conditions are displayed. The figure shows that the difference was related to increased brain activation in good play trials and decreased brain activation in poor play ones.


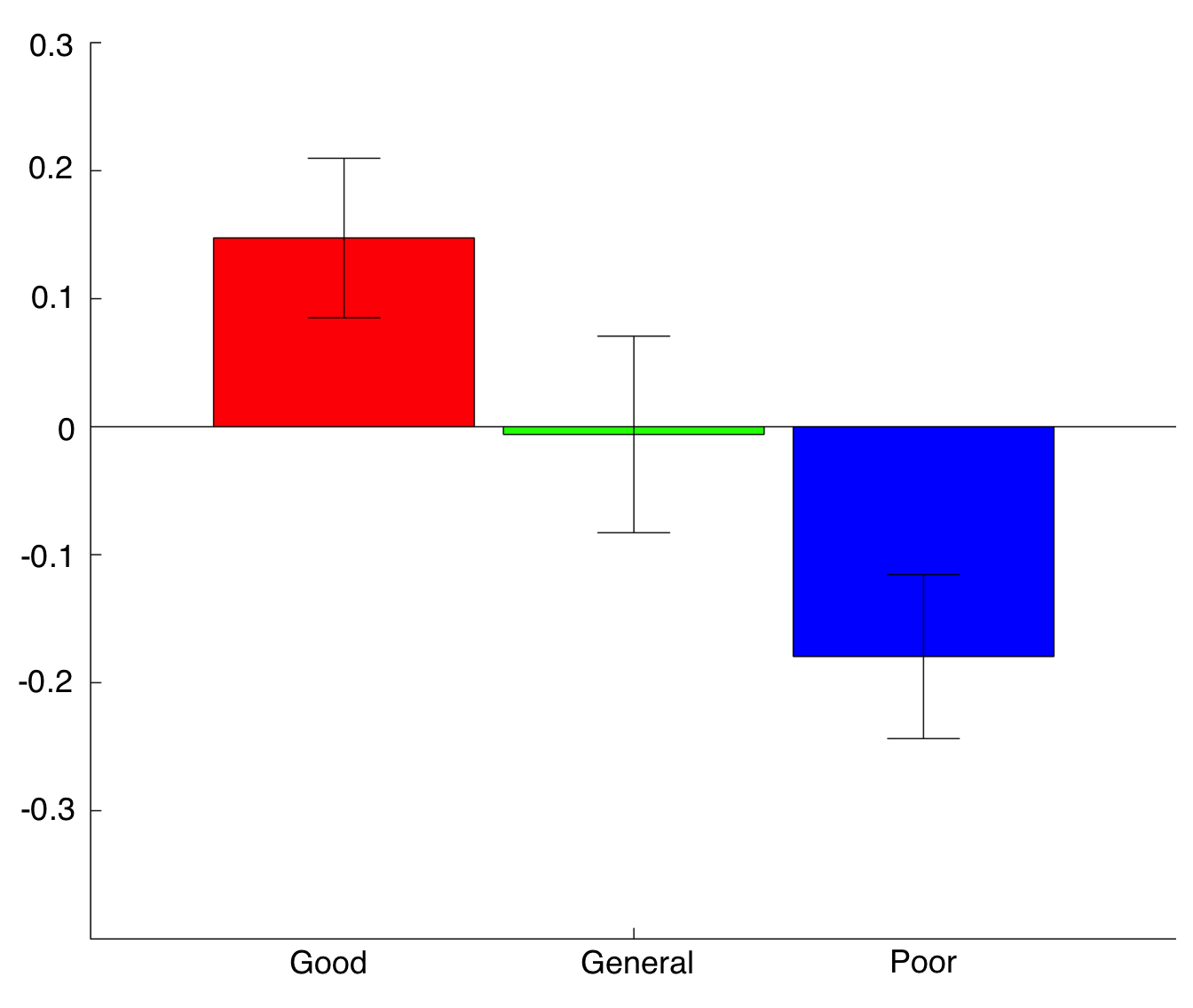


**Supplementary Figure 4:** Beta weights representing activation of the inferior frontal gyrus in the different conditions are displayed. The figure shows the difference was related to increased brain activation in good play trials and decreased brain activation in poor play ones.


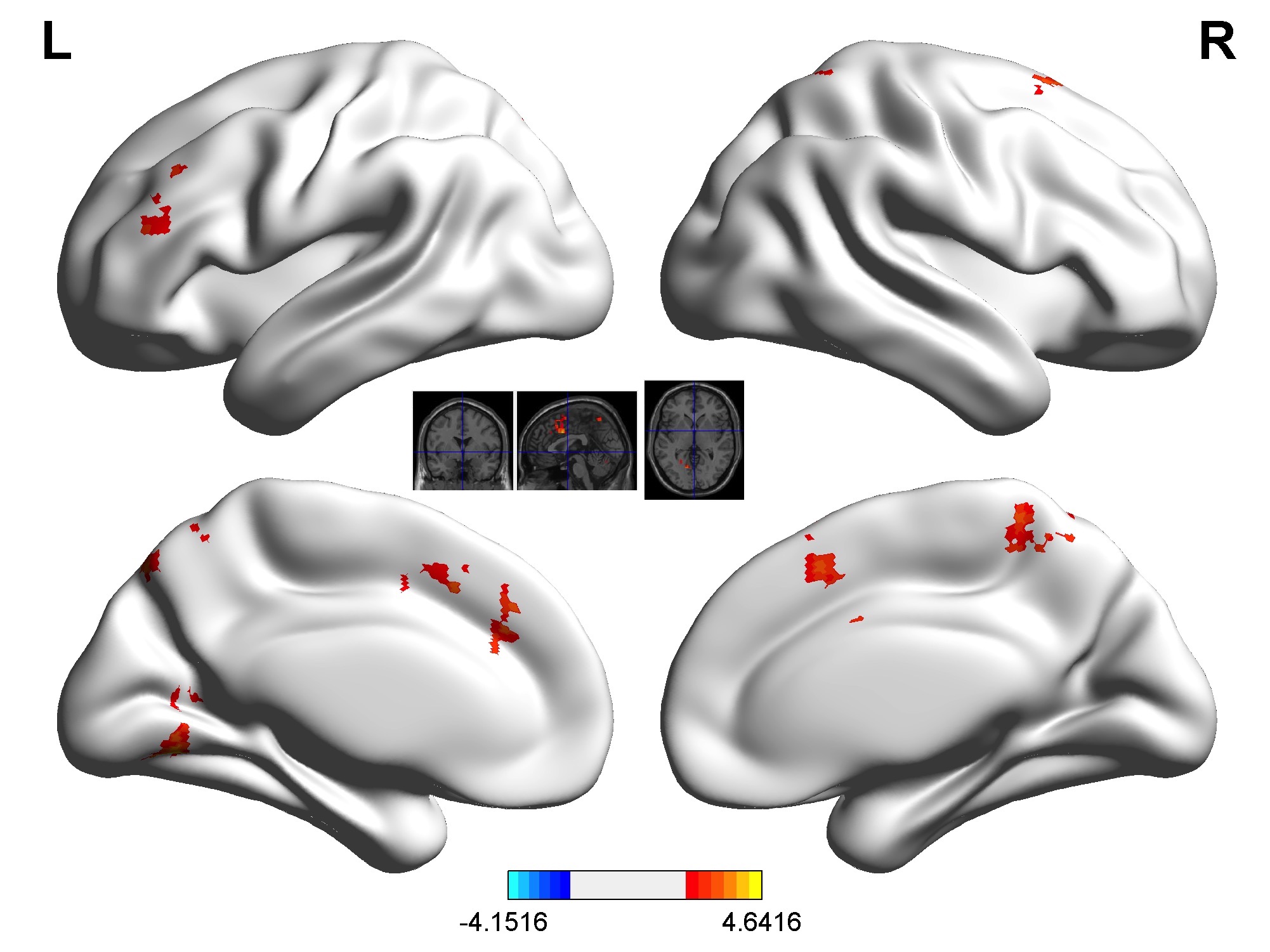


**Supplementary Figure 5**: Functional connectivity between the declive and the other brain regions in good-average comparison.

**Supplementary Table 1: Functional connectivity between the declive and other brain regions in the good-average comparison**

| Cluster Number | x,y,z^a^ | Peak Intensity | Cluster Size^b^ | Region ^c^ | Brodmann’s Area |
| --- | --- | --- | --- | --- | --- |
| 1 | -18,-66,-9 | 4.419 | 124 | Lingual Gyrus | 17,19 |
| 2 | -48,39,18 | 3.660 | 143 | L Middle Frontal Gyrus | 9,10,46 |
| 3 | 0,9,36 | 4.033 | 142 | Cingulate Gyrus | 23,24 |
| 4 | -3,-60,60 | 3.357 | 138 | L Precuneus | 7 |

^a^ Peak MNI Coordinates. ^b^ Number of voxels. AlphaSim FWE correction *p*<0.01 with 120 contiguous voxels. Voxel size=3*3*3. ^c^ The brain regions were referenced to the software Xjview (<http://www.alivelearn.net/xjview8>) and verified through comparisons with a brain atlas.


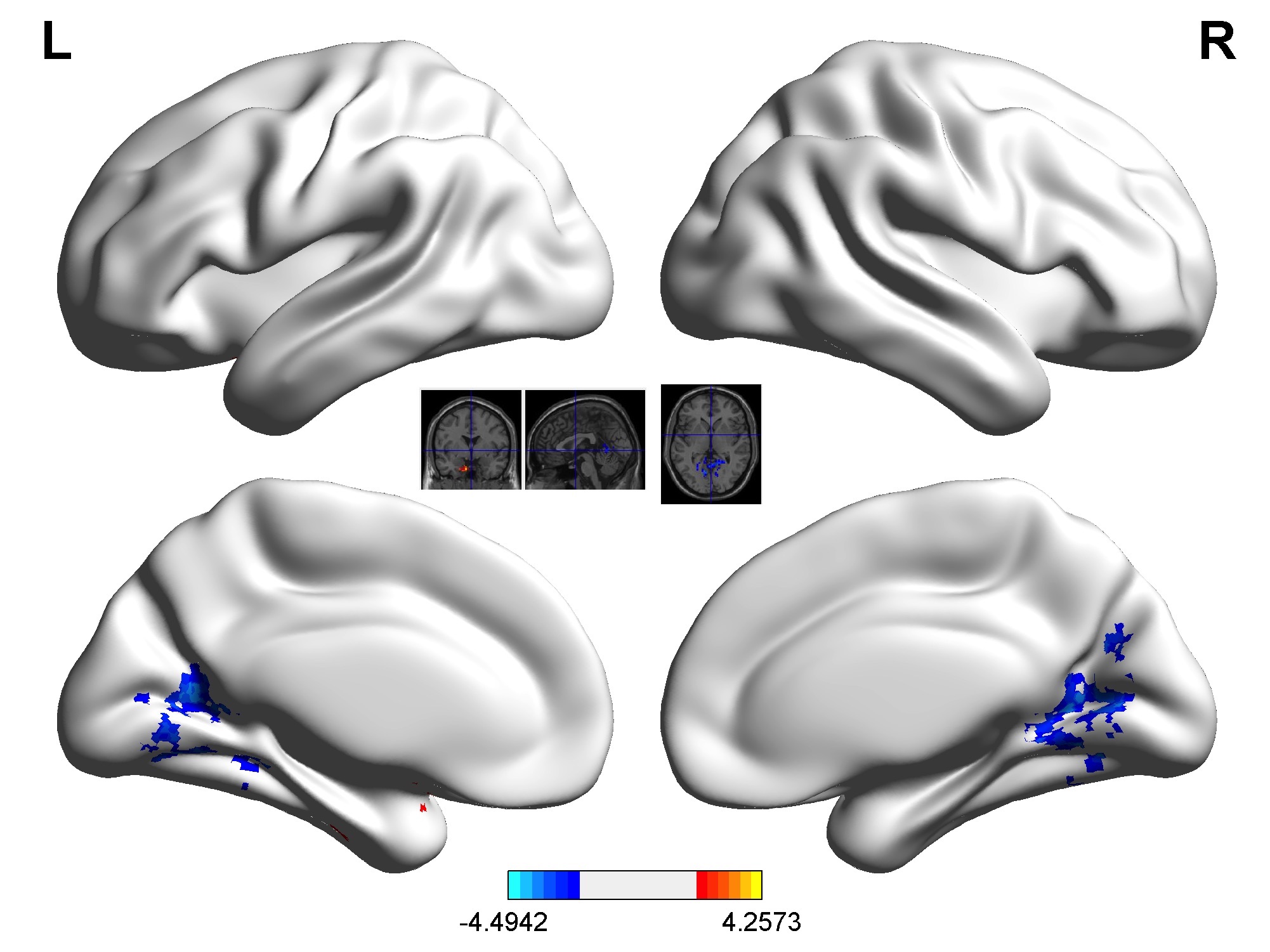


**Supplementary Figure 6:** Functional connectivity between the declive and the other brain regions in the average-poor comparison.

**Supplementary Table 2: Functional connectivity between the declive and other brain regions in the average-poor comparison**

| Cluster Number | x,y,z^a^ | Peak Intensity | Cluster Size^b^ | Region ^c^ | Brodmann’s Area |
| --- | --- | --- | --- | --- | --- |
| 1 | -9,0,-33 | 3.953 | 124 | L Parahippocampal Gyrus / Uncus | 36 |
| 2 | 24,-72,24 | -4.494 | 567 | Posterior Cingulate/ Sub-Gyral | 23,31 |

^a^ Peak MNI Coordinates. ^b^ Number of voxels. AlphaSim FWE correction *p*<0.01with 120 contiguous voxels. Voxel size=3*3*3. ^c^ The brain regions were referenced to the software Xjview (<http://www.alivelearn.net/xjview8>) and verified through comparisons with a brain atlas.


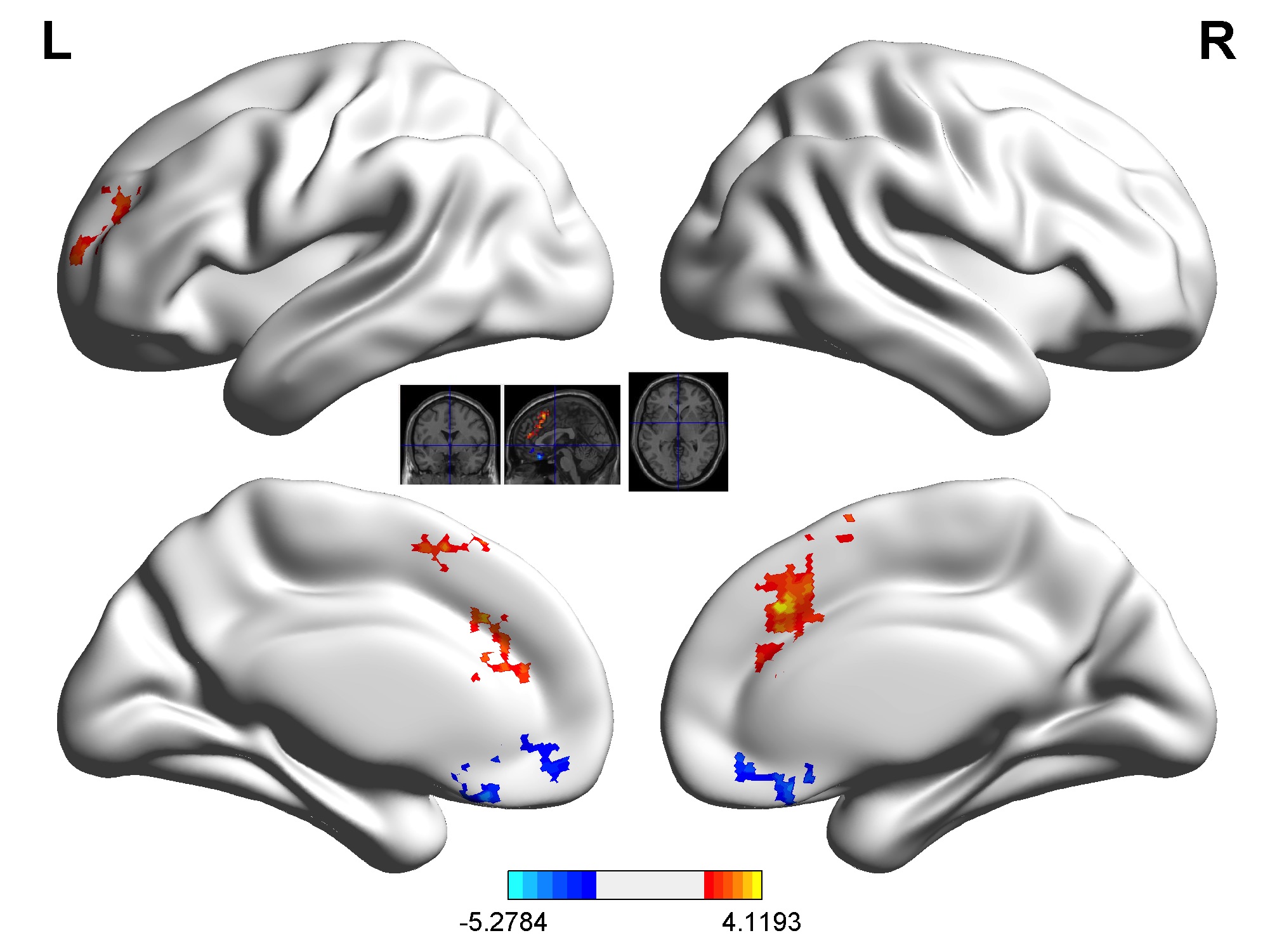


**Supplementary Figure 7:** Functional connectivity between the precentral gyrus and other brain regions in the good-average comparison.

**Supplementary Table 3: Functional connectivity between the precentral gyrus and other brain regions in the good-average comparison**

| Cluster Number | x,y,z^a^ | Peak Intensity | Cluster Size^b^ | Region ^c^ | Brodmann’s Area |
| --- | --- | --- | --- | --- | --- |
| 1 | 0,27,-21 | -5.278 | 136 | Medial Frontal Gyrus | 8,9 |
| 2 | 3,27,39 | 4.077 | 497 | Superior Frontal Gyrus/ Medial Frontal Gyrus | 4,6,8,9 |

^a^ Peak MNI Coordinates. ^b^ Number of voxels. AlphaSim FWE correction *p*<0.01with 120 contiguous voxels. Voxel size=3*3*3. ^c^ The brain regions were referenced to the software Xjview (<http://www.alivelearn.net/xjview8>) and verified through comparisons with a brain atlas.


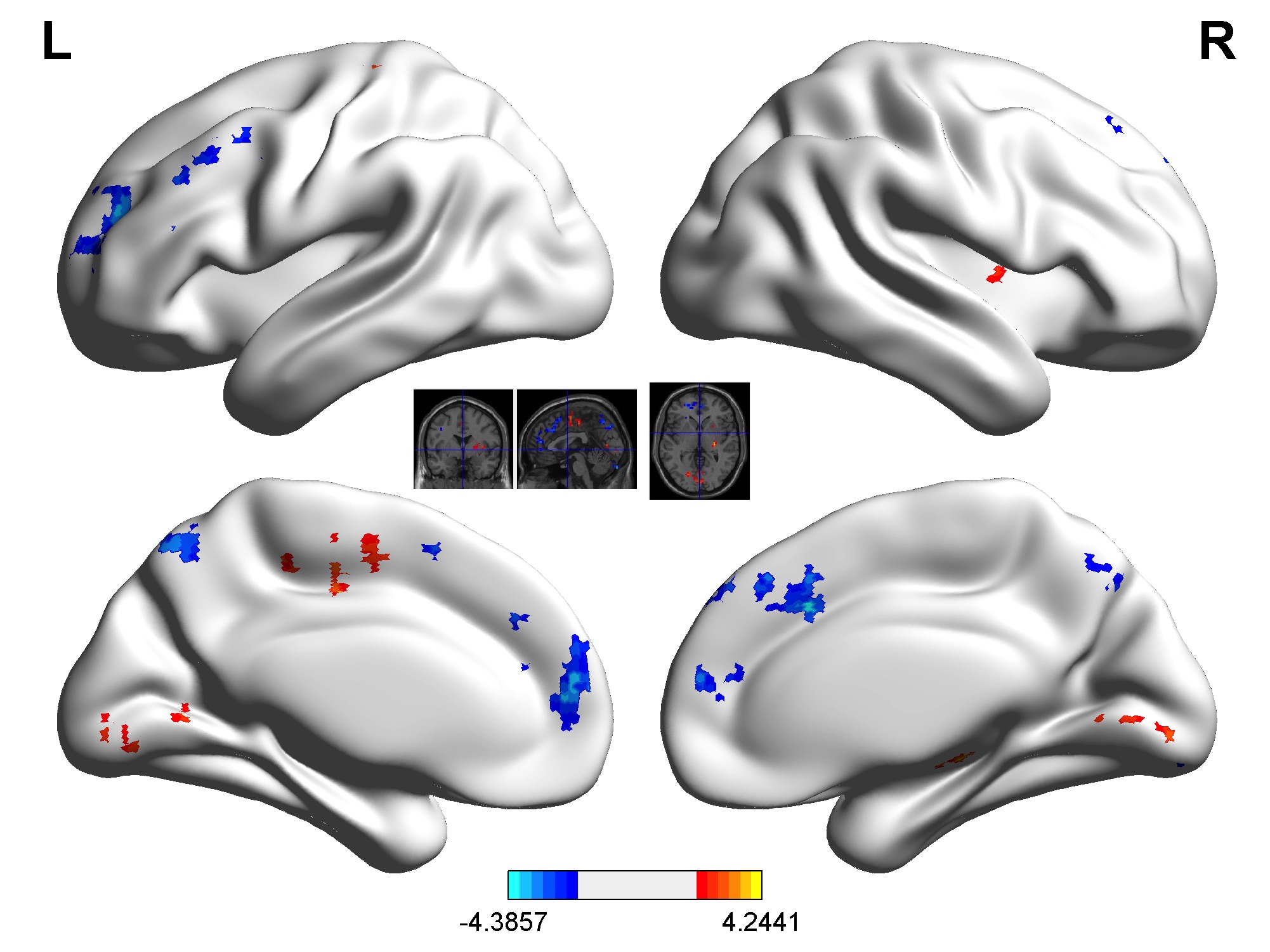


**Supplementary Figure 8:** Functional connectivity between the precentral gyrus and other brain regions in the average-poor comparison.

**Supplementary Table 4: Functional connectivity between the precentral gyrus and other brain regions in the average-poor comparison**

| Cluster Number | x,y,z^a^ | Peak Intensity | Cluster Size^b^ | Region ^c^ | Brodmann’s Area |
| --- | --- | --- | --- | --- | --- |
| 1 | 6,-84,-18 | -3.842 | 121 | Declive/ Pyramis |  |
| 2 | 27,-21,0 | 4.065 | 133 | Sub-lobar/Extra-Nuclear |  |
| 3 | 6,-75,6 | 3.304 | 127 | Cuneus/Lingual Gyrus | 17, |
| 4 | -9,54,12 | -3.937 | 305 | Medial Frontal Gyrus | 8,9 |
| 5  6  7  8 | -36,21,24  -9,-18,42  9,21,36  -6,-66,57 | -3.308  4.244  -4.008  -3.725 | 138  234  170  143 | Middle Frontal Gyrus  Medial Frontal Gyrus  Medial Frontal Gyrus/  Precuneus | 9,10,46  8,9  8,9,22,23,24  7 |

^a^ Peak MNI Coordinates. ^b^ Number of voxels. AlphaSim FWE correction *p*<0.01with 120 contiguous voxels. Voxel size=3*3*3. ^c^ The brain regions were referenced to the software Xjview (<http://www.alivelearn.net/xjview8>) and verified through comparisons with a brain atlas.


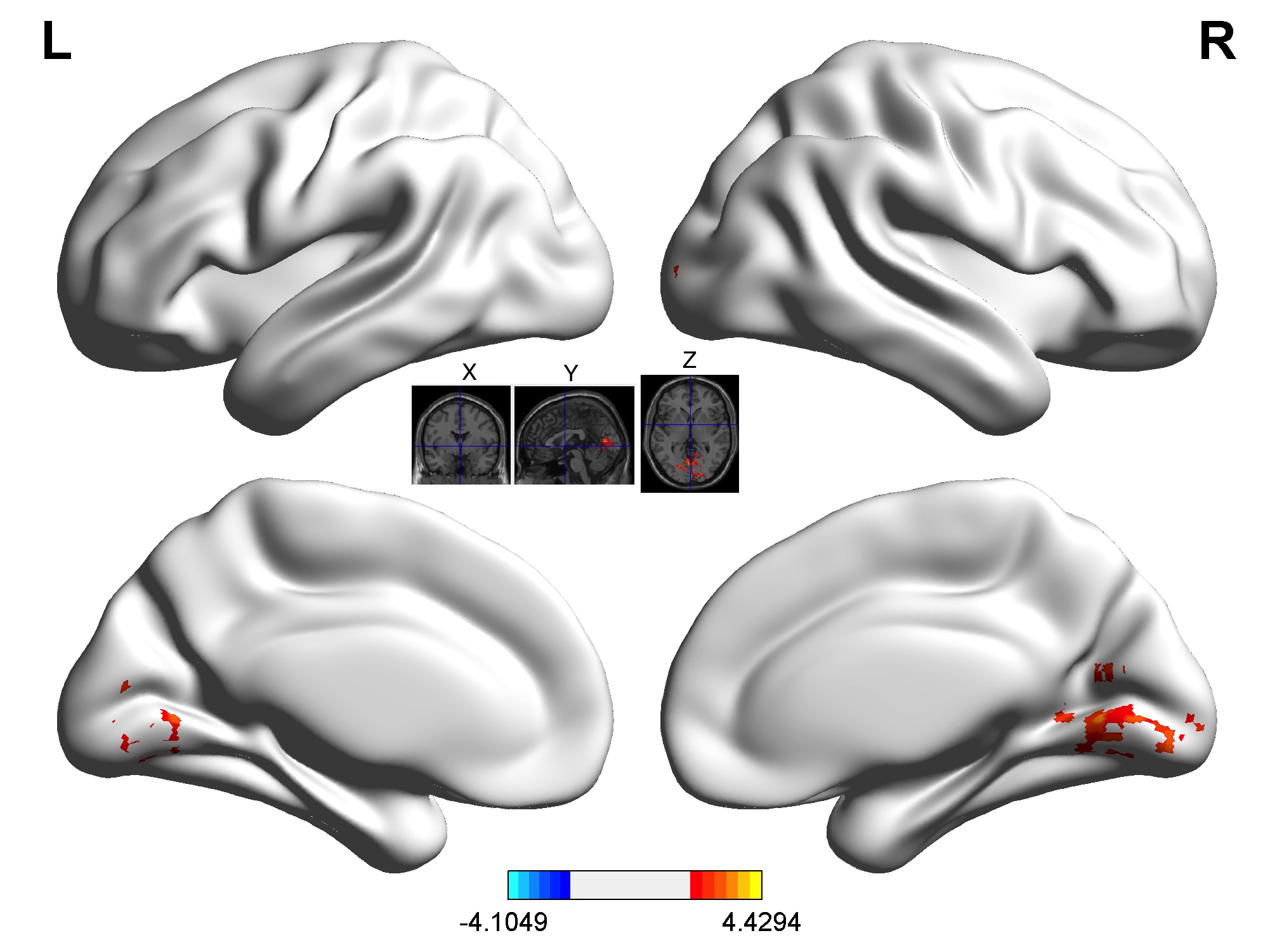


**Supplementary Figure 9:** Functional connectivity between the striatum and other brain regions in the good-average comparison.

**Supplementary Table 5: Functional connectivity between the striatum and other brain regions in the good-average comparison**

| Cluster Number | x,y,z^a^ | | Peak Intensity | | Cluster Size^b^ | | Region ^c^ | | Brodmann’s Area | |  |
| --- | --- | --- | --- | --- | --- | --- | --- | --- | --- | --- | --- |
| 1 | | 18,-93,3 | | 4.429 | | 387 | | Lingual Gyrus/ Cuneus | | 17 | |

^a^ Peak MNI Coordinates. ^b^ Number of voxels. AlphaSim FWE correction *p*<0.01with 120 contiguous voxels. Voxel size=3*3*3. ^c^ The brain regions were referenced to the software Xjview (<http://www.alivelearn.net/xjview8>) and verified through comparisons with a brain atlas.


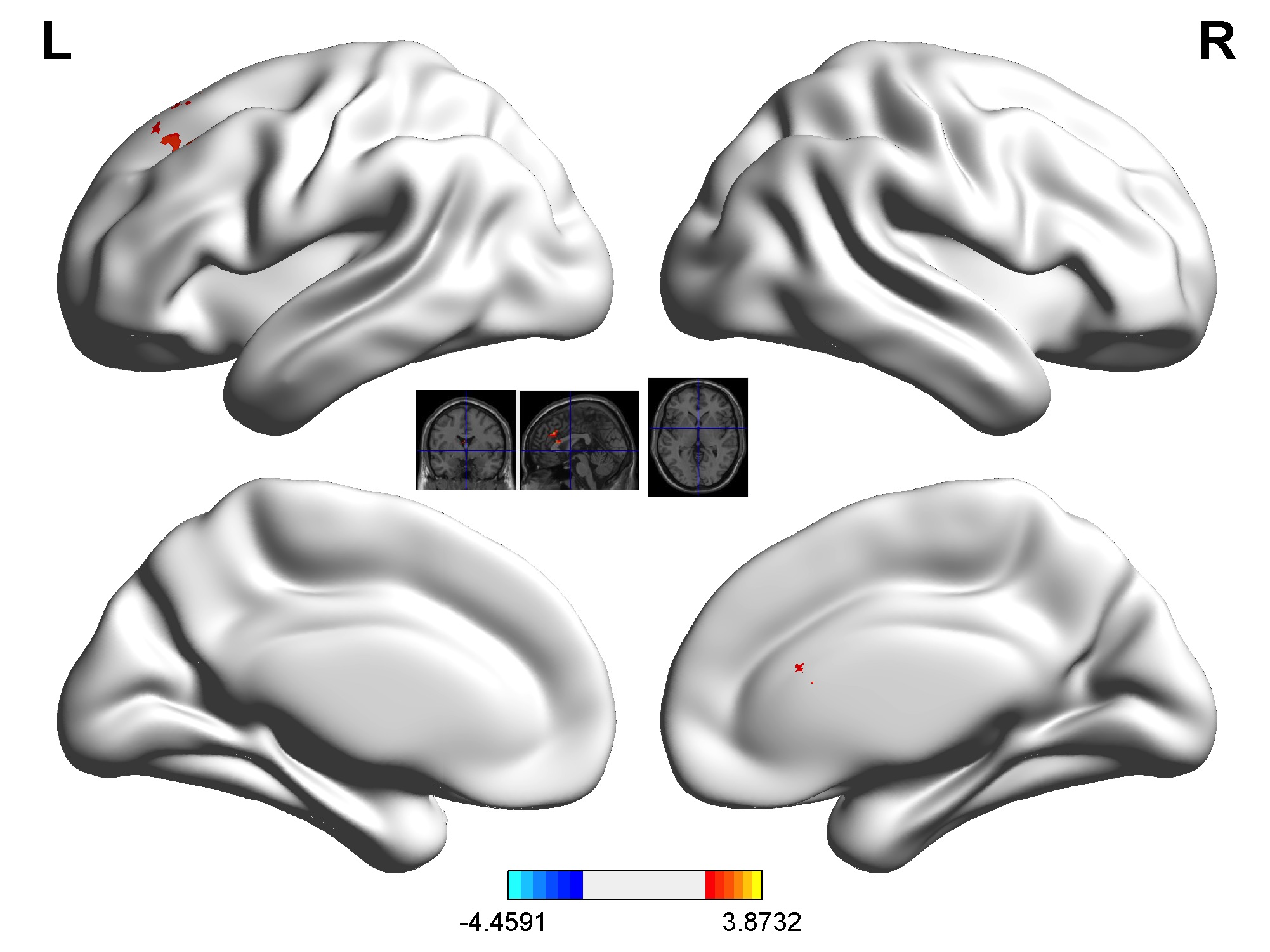


**Supplementary Figure 10:** Functional connectivity between the striatum and the other brain regions in the average-poor comparison.

**Supplementary Table 6: Functional connectivity between the striatum and other brain regions in the average-poor comparison**

| Cluster Number | x,y,z^a^ | | Peak Intensity | | Cluster Size^b^ | | Region ^c^ | | Brodmann’s Area | |
| --- | --- | --- | --- | --- | --- | --- | --- | --- | --- | --- |
| 1 | | -3,24,18 | | 3.619 | | 220 | | Middle Frontal Gyrus / Sub-lobar | | 9,10,46 |

^a^ Peak MNI Coordinates. ^b^ Number of voxels. AlphaSim FWE correction *p*<0.01 with 120 contiguous voxels. Voxel size=3*3*3. ^c^ The brain regions were referenced to the software Xjview (<http://www.alivelearn.net/xjview8>) and verified through comparisons with a brain atlas.
